# Effects of application of phosphate and phosphate-solubilizing bacteria on bacterial diversity and phosphorus fractions in a Phaeozems

**DOI:** 10.1016/j.heliyon.2023.e22937

**Published:** 2023-11-26

**Authors:** Yu Tang, Yan-Jing Che, Xue-Yan Bai, Zi-Ying Wang, Si-Yu Gu

**Affiliations:** College of Resource and Environment, Northeast Agricultural University, Harbin, 150030, China

**Keywords:** Environmental microorganisms, P utilization, P conversion, Correlation analysis, Structural equation model

## Abstract

The aim of this study is to improve the utilization of phosphorus (P) in soil, and to study the effects of phosphate-solubilizing bacteria (PSB) on P fractions and bacterial communities. In this experiment, we reduced the amount of P fertilizer by 30 % and 40 % respectively to studied the effects of combined application of bacterial fertilizers on soil microbial community and phosphate transformation process under different fertilization rates. The results showed that the application of PSB affected the transformation process of different P fractions. PSB had the most significant impact on organic phosphorus (*p* < 0.05). Correlation analysis showed that the abundance of bacteria was significantly correlated to the P fractions, indicating that the application of PSB had affected the bacterial community structure. In addition, Structural Equation Model (SEM) analysis showed that there was a causal relationship between the various visual variables. SEM confirmed the response relationship between bacterial communities and P components. Based on these results, we concluded that the application of PSB increased the sensitivity of P components, especially Olsen-P and MBP, to soil microorganisms. The application of PSB is an effective method to improve P utilization.

## 1 Introduction

Phosphorus (P) is one of the essential elements in plant growth. After being applied to the soil, it is easily utilization in large quantities [1]. Therefore, to ensure the P required for plant growth, phosphate fertilizer is also one of the most commonly used fertilizer types in agricultural production [2].

Phosphorus applied to soil is easily fixed by the soil, causing it to accumulate in large quantities in soil [3]. The insoluble phosphorus formed after accumulation is difficult to be directly absorbed and utilized by plants. Therefore, in greenhouse agricultural production, the blind application of a large amount of phosphorus fertilizer is severe. The application amount of phosphate fertilizer is 5–20 times the fertilizer required by plants [4]. Long-term excessive use of phosphate fertilizer can enrich a large amount of P in the 0–20 cm plow layer. Moreover, it can also lead to the release of P in deep soil layers of 20–40 cm [5]. Even though P fertilizer is very necessary in soil, most of P fertilizer applied to soil quickly combines with iron, aluminum, calcium, magnesium, etc. in soil, forming insoluble P that are difficult to be directly absorbed and utilized by plants, resulting in low P fertilizer utilization efficiency. Also, excessive release of P can lead to eutrophication of adjacent water bodies [6], one of the essential sources of agricultural non-point source pollution [7]. Therefore, in order to ensure the normal P requirements of plants and make full use of the insoluble P in the soil, it is urgent to repair the threat of excessive P to the environment [8]. Bioremediation is one of the most effective methods to improve the utilization of P [9].

Phosphate-solubilizing bacteria (PSB) play a crucial role in enhancing the utilization of P through biological pathway. A large number of studies have documented the research of PSB in water treatment, and there are few studies on the application of PSB in soil. In addition, biological phosphorus removal technology is rarely reported on the conversion of soil phosphorus, so this experiment will do some attempts and discussions in this part. In soil research, the phosphorus solubilization mechanism of phosphorus solubilizing microorganisms can usually be achieved through organic acids, proton exchange, complexation, and other pathways to dissolve and release insoluble phosphates [3]. And it is a microorganism that can convert insoluble phosphate into a form that can be absorbed by plants [10]. Many factors affect PSB, such as pH, temperature, carbon to P ratio [[11], [12], [13]]. Previous studies have shown that PSB can excrete insoluble phosphorus from the rhizosphere environment by secreting organic acids, proton exchange, and recombination. Research has shown that there is a large amount of inorganic phosphorus in soil, and the microbial phosphatase and inositol hexaphosphatase are important factors affecting the release of soil organic phosphorus [14]. In this study, to clarify the mechanism of PSB in soil, we proposed adding different concentrations of PSB consortium to adjust and control the characteristics of the soil in the greenhouse. Explore the impact of P fertilizer combined with PSB application on soil physicochemical properties, microbial community structure, and the response relationship between soil physicochemical properties and microbial community structure, and determine the key factors affecting soil P transformation. Based on this, a method was proposed to replace partial P fertilizer with PSB to regulate soil properties, filling the gap in the application of PSB in soil and providing theoretical support and technical guidance for the rational use of P fertilizer in the future.

On this basis, the following aspects of research were carried out: (i) The dynamic evolution of soil P forms during plant growing; (ii)Comparisons were made between the interrelationships between various P components and microorganisms; (iii) Exposed the potential correlations between the P components. These would be a way to reduce P loss and increase P utilization efficiency.

## 2 Materials and methods

### 2.1 Plant growing process and sample collection

Conducted greenhouse cultivation pots experiments using peppers (F10-8710). The peppers were grown in the black soil in the Heilongjiang Academy of Agricultural Sciences of China. The black soil selected for the experiment is classified as Phaeozems in World Reference Base [15]. The physical and chemical characteristics of soil included the followings: PH is 6.40 ± 0.05, total phosphorus (TP) is 0.27 g/kg, Olsen phosphorus (Olsen-P) content is 7.24 mg/kg, organic phosphorus (OP) content is 268.21 mg/kg, soil organic carbon (SOC) is 18.82 g/kg, total nitrogen (TN) content is 1.26 g/kg (Table 1). The planting pot used in this experiment was 34.0 cm in diameter and 25.0 cm in height. We applied 3 kg of black greenhouse soil in each pot. Two 15-day seedlings of peppers were planted per pot. Added 400 ml of water to each pot every two days. Phosphate fertilizer and PSB consortium were added as base fertilizers on the first day, without topdressing in later stage. Potassium sulfate compound fertilizer (N: P: K = 12:18:15) was selected in this experiment, which met the national standard DB15063-94. After 10 generations of subculture, the PSB strain was inoculated into a triangular flask containing 100 mL of seed medium (glucose 10 g, (NH_4_)_2_SO_4_ 0.5 g, NaCl 0.3 g, MgSO_4_·7H_2_O 0.3 g, KCl 0.3 g, MnSO_4_·4H_2_O 0.03 g, FeSO_4_·7H_2_O 0.03 g, KH_2_PO_4_ 0.2 g, distilled water 1000 mL, pH 7.0–7.2), and incubated in a rotating shaker at 40 °C and 180 rpm for 48 h [16,17]. The preparation of PSB consortium in this experiment was as follows: (1) Centrifuged and concentrated the cultivated PSB bacterial strains, adjusted the pH of the concentrated bacterial culture medium to 4.0, added 3 wt ‰ sodium benzoate, mixed well for later use; (2) Added corn powder evenly according to the solid-liquid ratio (g/mL) of 1:2, mixed it evenly, and placed it on a constant temperature shaker and shake it at 37 °C for 1h at the speed of 50 r/min; (3) Dried the obtained solid bacterial agent in a constant temperature oven at 35 °C to a moisture content of 8 wt%; (4) Added 1.5 wt ‰ iron powder deoxidizer to the solid bacterial agent obtained above, mixed evenly and placed it at room temperature, seal and store for future use; (5) The deoxidizer was preferably a mixture of reducing iron powder, activated carbon, and sodium chloride mixed in a mass ratio of 8:2:1 [18]. After activation of the PSB consortium, the number of colonies calculated by coating the plate was 3.2 × 10^7^ CFU/g. On this basis, this experiment referred to the actual amount of fertilizer applied by the Academy of Agricultural Sciences, and gradually reduced the amount of phosphate fertilizer applied to the PSB consortium. Among them, the control group (F) only applied 30 g of phosphorus fertilizer per pot. In the 30 % substitution of phosphate fertilizer by PSB consortium group (F30), the phosphate fertilizer application amount was 21 g per pot, and the PSB consortium amount was13 g per pot. The 40 % substitution of phosphate fertilizer by PSB consortium group (F40) phosphate fertilizer application amount was 18 g per pot, and the PSB consortium amount was 17 g per pot. Besides, F30 and F40 used straw as carbon source. The soil sampling time was determined based on the growth cycle of peppers. Selected the germination stage, seedling stage, initial flowering stage, and fruit setting stage corresponding to the growth of peppers for 3, 47, 77, and 157 days. P fractions and microbial analysis have been reported in previous studies. Soil pH was measured using a 1:2.5 soil water ratio. Total phosphorus (TP) in soil was determined by molybdenum-antimony resistance colorimetry after HClO_4_–H_2_SO_4_ digestion [19]. Organic phosphorus (OP) was determined by measuring the difference between the 550 °C calcination for 1 h and the unburned value of H_2_SO_4_ extraction [19]. Olsen phosphorus (Olsen-P) was determined using 0.5 mol L^−1^ NaHCO_3_ extraction method [19]. Microbial biomass phosphorus (MBP) was using chloroform fumigation method [20,21]. All the experiments were independently repeated with three replications.

**Table 1:** Physical and chemical indicators of experimental soil.

SOC	TN	TP	pH	Olsen-P	OP
(g kg^−1^)				(mg kg^−1^)	
18.82 ± 3.08a	1.26 ± 0.52b	0.27 ± 0.08a	6.40 ± 0.05a	7.24 ± 1.25a	268.21 ± 89.67a

### 2.2 Statistical analysis

Performed one-way ANOVA and *t*-test using software SPSS 22.0. Used Origin pro8.0 to draw the P components characteristics diagram [22]. Correlation analysis was plotted in R. Linear and quadratic regression analysis was performed for different phosphorus components and microbial data. The maximum results of adjusted R^2^ were shown and the ggplot2 package was used to draw the results. Statistical analysis was performed, and a *p*-value of less than 0.05 was considered significant, while a *p*-value of less than 0.01 was considered highly significant. The confidence interval of the regression curve was 95 % [23]. NMDS was the statistical result of the principal coordinate analysis on the bacterial community. The structural equation model (SEM) was constructed using the maximum likelihood estimation method by AMOS 21.0 software (IBM Corporation Software Group, Somers, NY). Non-significant X^2^ test (p > 0.05), goodness-of-fit index (GFI>0.90) and root-mean-square error of approximation (RMSEA<0.05) were used to represent the overall goodness of fit [24,25]. Throughout this study, statistical significance was consistently maintained at *p* < 0.05.

## 3 Results and discussion

### 3.1 Changes of P fractions during plant growing

**Fig. 1:**
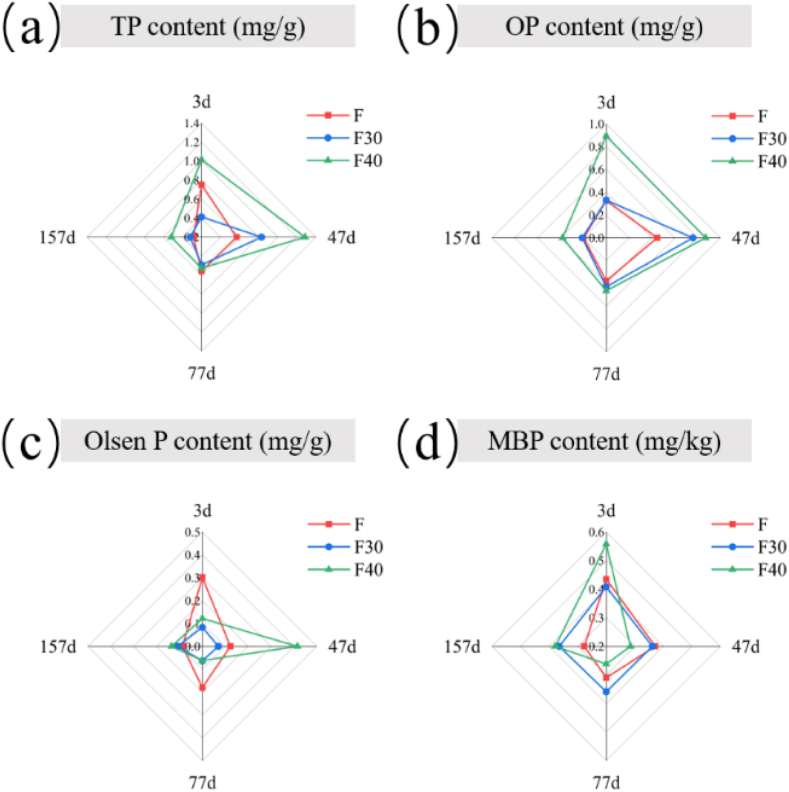
Changes of total phosphorus (TP, a), organic phosphorus (OP, b), Olsen phosphorus (Olsen-P, c), microbial biomass phosphorus (MBP, d) during plant growing.

The concentrations of various P components were depicted in Fig. 1. Total phosphorus (TP) is the total amount of phosphorus in soil. The total phosphorus (TP) content of the soil generally decreased, except for the F30 group, which increased on the 47th day. This result may be due to the degradation of organic components (such as straw, animal and plant residues) in soil during the application process, which caused the release of P in the soil [26]. From Fig. 1a, it was obviously that on the 47th day of growing, the TP content of F30 and F40 was significantly higher than that of F (*p* < 0.05). And could provide more nutrients for plant growth in the later stage of growing. The phosphorus contained in organic phosphides such as phospholipids, nucleic acids, and phytochemicals in soil is named soil organic phosphorus (OP). The results showed that the organic phosphorus (OP) content of all treatment groups increased first and then decreased (Fig. 1b). During the whole growing process, the OP content of F30 and F40 always kept a high level. On the 47th day, the OP content of F30 and F40 was significantly higher than that of group F (*p* < 0.05). This result indicated that the inoculation of PSB promoted the formation of OP. Olsen phosphorus (Olsen-P) is easily absorbed and utilized by plants. Results in Fig. 1c showed that the content of Olsen phosphorus (Olsen-P) in F40 increased significantly in the early stage of growing (3-47d) (*p* < 0.05) (Fig. 1c). This result showed that PSB consortium can promote the release of soil Olsen-P [27]. Soil microbial biomass phosphorus (MBP) refers to the phosphorus contained in all living microorganisms in soil. As shown in Fig. 1d, although the microbial biomass phosphorus (MBP) content of F was basically the same as the MBP content of F30 on the third day, the MBP content of F showed a downward trend as the application time extended, while the MBP content of the F30 group remained stable throughout the process. In summary, the application of PSB was a feasible measure to promote the conversion of P, providing the necessary P source for plant growth.

### 3.2 Relationships among P fractions and bacterial communities

**Fig. 2:**
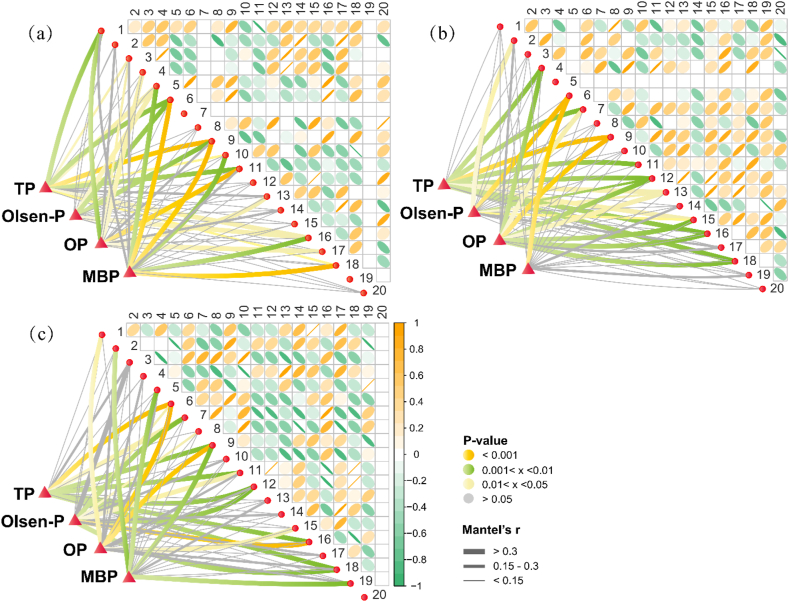
Correlation analysis between P fractions and bacterial communities in different treatments are shown, with a color gradient denoting Spearman's correlation coefficient. Edge width corresponds to the Mantel's r statistic for the corresponding distance correlations, and edge color denotes the statistical significance based on 9999 permutations.

In order to compare the relationship between the microbial community and the P fractions in F, F30, and F40, we correlated the gene abundance of the microbial band with the P fractions factor (Fig. 2) [28]. Overall, microorganisms were closely related to the P fractions. There was no band 7 and band 19 in F (Fig. 2a), but band 7 was derived from PSB and existed in F30 and F40. This indicated that PSB had successfully integrated into the soil and survived. Band 7 responded most closely to TP and OP in F30 (0.01< *p* < 0.05) (Fig. 2b). As shown in Fig. 2c, band 7 had the closest response to TP and Olsen-P in F40 (0.01< *p* < 0.05). These results indicated that band 7 was always closely related to TP. In general, the microbial activity of F had the strongest correlation with MBP, and in F30 and F40, microbial activity was always closely related to TP and OP. Overall, F40 had the highest number of bands, with the strongest correlation between P components and bands, indicating that the more P fertilizer was replaced by a large number of microorganisms, the closer the relationship between P components. This was consistent with the study by Wei et al. that the relationship between microbial networks under P deficiency stress was more complex and interspecific competition was intense [29]. In summary, due to the synergy and antagonism between the microbial populations, the administration of PSB consortium led to changes in the microbial population structure. It also be said that the application of PSB could change the microenvironment of the soil, thereby changing the product yield of soil P conversion and related processes.

### 3.3 Possible linkages among different P fractions

**Fig. 3:**
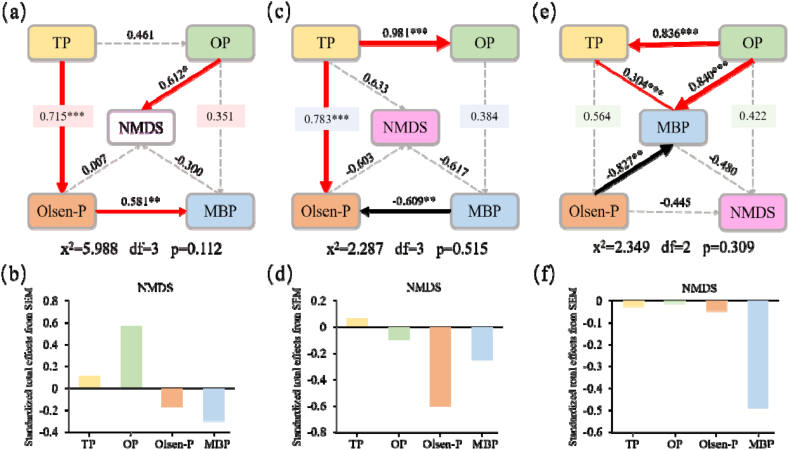
Possible linkages among different N fractions. (a), (c), (e) SEM of P fractions and bacterial communities during plant growing in different treatment; (b), (d), (f) standard total effects (direct plus indirect effects) of NMDS on TP, OP, Olsen-P and MBP in different treatment. NMDS represents the data derived from the principal coordinate analysis of bacteria community. Solid and dashed arrows indicate significant (*p* < 0.05) and non-significant relationships (*p* > 0.05), respectively. Significance levels are indicated: ∗ *p* < 0.05, ∗∗ 0.001 < *p* < 0.05, ∗∗∗ *p* < 0.001.

SEM was a confirmatory model that verifies the assumed causal relationship between variables through data fitting [30]. In this way, we could have a more direct understanding of the relationship between various P fractions and microorganisms. From Fig. 3a, it could be seen that TP had a positive effect on Olsen-P in F (λ = 0.715，*p* < 0.001). Then Olsen-P exerted a directly positive impact on MBP(λ = 0.581，0.001< *p* < 0.05).This result showed that TP affects the change of MBP by affecting Olsen-P. The reduction of TP lead to the reduction of soil MBP, which also confirmed the findings of previous studies (Fig. 1). Fig. 3b showed the standard total impact of NMDS on TP, OP, Olsen-P, MBP. The positive impact on OP was the greatest (λ = 0.567), and the standard total impact of NMDS on TP was 0.114. From Fig. 3c, it could be seen that TP had a positive effect on the OP of F30(λ = 0.981，*p* < 0.001). TP also had a significant positive effect on Olsen-P (λ = 0.783，*p* < 0.001), but MBP exerted a directly negative impact on Olsen-P(λ = −0.609，0.001< *p* < 0.05). The NMDS had the greatest positive impact on the P fractions was TP (λ = 0.067), and the greatest negative impact was Olsen-P (λ = −0.603), followed by MBP (λ = −0.250) (Fig. 3d). It showed that in F30, the correlation between TP and microorganisms was the closest, which also verified the results of previous studies (Fig. 2b). In F40, OP had a direct positive effect on TP (λ = 0.836, *p* < 0.001), OP can also directly affect MBP and indirectly affect TP (Fig. 3e). Conversely, raised Olsen-P levels were found to restrained the formation of MBP. This could possibly be attributed to the increased influence of the microbial community influenced by Olsen-P, indirectly impacting the formation of MBP. Fig. 3f showed that MBP had the greatest impact on the P fractions of NMDS (λ = −0.488). It showed that MBP in F40 is susceptible to microorganisms. This was consistent with the findings of Fig. 2c. As shown in Fig. 3d and f, applying PSB consortiums increased the sensitivity of Olsen-P and MBP to soil microorganisms. Among them, Olsen-P, as a phosphorus that was easily absorbed and utilized by plants, had a key impact on promoting plant growth. In summary, the mixed application of PSB and phosphate fertilizer had a significant effect on regulating the conversion of soil P fractions, and the efficiency of P utilization can be improved to achieve the purpose of reducing fertilizer application and rational use of fertilizer.

### 3.4 A novel perspective to improve the utilization of P during plant growth

**Fig. 4:**
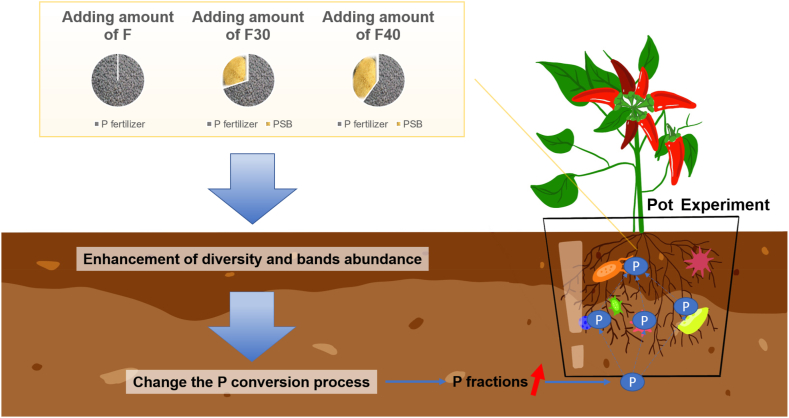
Conceptual diagram of changes in the interaction between P and bacterial communities in the soil after the PSB consortium is applied to the soil.

This experiment availably improved the availability of P increased in soil due to fertilization residues, which not only continuously provided a large amount of available P for crop growth, but also avoided the eutrophication of water bodies caused by P runoff, which harmed the environment. Therefore, this study provides a novel perspective to improve the utilization of P during plant growth (Fig. 4). Firstly, by applying a certain amount of PSB to the soil, it can effectively improve the content of P in the soil. The MBP in the soil can reflect the microbial content in the soil to a certain extent. Because PSB can dissolve a large amount of insoluble P through biological pathways and be absorbed and utilized by plants [31]. Therefore, applying PSB in the soil can increase the content of OP, MBP, and TP in the soil. And strengthen the relationship between some microbial flora in the soil and its correlation. Secondly, in order to fully realize the efficient use of PSB, it is necessary to further study the role of different types of soil mineral elements and related microorganisms [32], and to clarify the specific mechanism of mineral transformation at each stage of growing [33]. Combining the analysis of the elemental components of each part of the plant, explore the root cause of the influence of PSB on the growth of the plant, and determine the key factors affecting the transformation of soil mineral elements, the structure of microbial populations, and plant growth [34]. Moreover, in order to gain a deeper understanding of the PSB mechanism of P dissolution, it is necessary to further study different soil environments. For soil that has already been excessively applied with phosphorus fertilizer, due to its high content of phosphorus and a high proportion of water-soluble phosphorus, phosphate accumulating organisms (PAOs) can be utilized to accumulate a large amount of polyphosphates in the metabolic mechanism of microorganisms. Combined with their application in sewage treatment [35], they can be made into new microbial fertilizers and combined with PSB microbial agents, Improve the biological migration and transformation of soil phosphorus, and change the existing forms of phosphorus in soil.

## 4 Conclusions

This study proposed a novel method to improve P utilization and microbial activity in the soil. Results showed that even if the application of phosphate fertilizer is reduced, the application of PSB increased the concentrations of part of the P fractions, especially the increase in OP was the most significant, which promoted the transformation of P. At the same time, utilized PSB could affect the microbial population structure and enhanced the interaction between some microorganisms (such as band 7) and phosphorus components. The SEM results indicated that there was a certain correlation between microorganisms in soil and phosphorus components. Therefore, PSB can effectively replace phosphorus fertilizer to achieve the effect of reducing fertilizer application and increasing efficiency, accelerating the transformation of agricultural production methods to environmentally friendly resource conservation, and promoting green and sustainable development of agriculture.

In this experiment, PSB consortiums were used to replace part of inorganic fertilizers, and the effect of PSB consortiums on soil phosphorus conversion and soil microorganisms were preliminarily revealed. This study is a preliminary short-term study to improve utilization efficiency, which is the limitation of this experiment. The next step of the work plan should be to further understand and predict the distribution changes of phosphorus components during the long-term planting process through mathematical kinetic model fitting. In future studies, the phosphorus solubilization mechanism of PSB in different soil environments can be discussed by adding the factor of organic fertilizer. In addition, in order to fully realize the efficient utilization of PSB, it is necessary to further study the roles of different types of soil mineral elements and related microorganisms, clarify the specific mechanism of mineral transformation at different stages of application. The effects of PSB on the growth state of plants were studied by analyzing the elements of each part of the plant. To determine the key factors affecting the transformation of soil mineral elements, microbial community structure and plant growth, solve the problem of PSB directional regulation, and reduce fertilizer planting for agricultural production. The mechanism by which phosphorus solubilizing microorganisms participate in the transformation of phosphorus components in soil has not yet been explained. This study only developed a basic model based on possible interaction relationships. The next step in research should be to construct a more detailed network map of phosphorus solubilization mechanisms of phosphorus solubilizing microorganisms through proteomics combined with metagenomic sequencing.

## Declaration of funding

This work is financially supported by the 10.13039/501100012166National Key Research and Development Program of China (2021YFD1500801).

## Data availability statement

All data, models, or code generated or used during the study are available from the corresponding author by request. (gusiyu@neau.edu.cn).

## CRediT authorship contribution statement

**Yu Tang:** Data curation, Formal analysis, Visualization, Writing – original draft, Writing – review & editing. **Yan-Jing Che:** Resources, Validation. **Xue-Yan Bai:** Software. **Zi-Ying Wang:** Methodology. **Si-Yu Gu:** Conceptualization, Funding acquisition, Investigation, Writing – review & editing.

## Declaration of competing interest

The authors declare that they have no known competing financial interests or personal relationships that could have appeared to influence the work reported in this paper.
