# Supplementary material for: Effects of application of phosphate and phosphate-solubilizing bacteria on bacterial diversity and phosphorus fractions in a Phaeozems
Source: Heliyon. 2023 Nov 26;9(12):e22937. doi: 10.1016/j.heliyon.2023.e22937 (PMC10716540; doi:10.1016/j.heliyon.2023.e22937)
Supplement: Multimedia component 1 [file mmc1.doc]

**Supplementary Information**

**Effects of application of phosphate and phosphate-solubilizing bacteria on bacterial diversity and phosphorus fractions in a Phaeozems**

Yu Tang^a^, Yan-Jing Che^a^,Xue-Yan Bai^a^, Zi-Ying Wang^a^, Si-Yu Gu^a, *^

a College of Resource and Environment, Northeast Agricultural University, Harbin 150030, China

* Corresponding author’s address: College of Resource and Environment, Northeast Agricultural University, Harbin 150030, China

Tel/Fax: +86 0451 55190927

E-mail address: gusiyu@neau.edu.cn

Supporting Information Includes:

- 1 figure


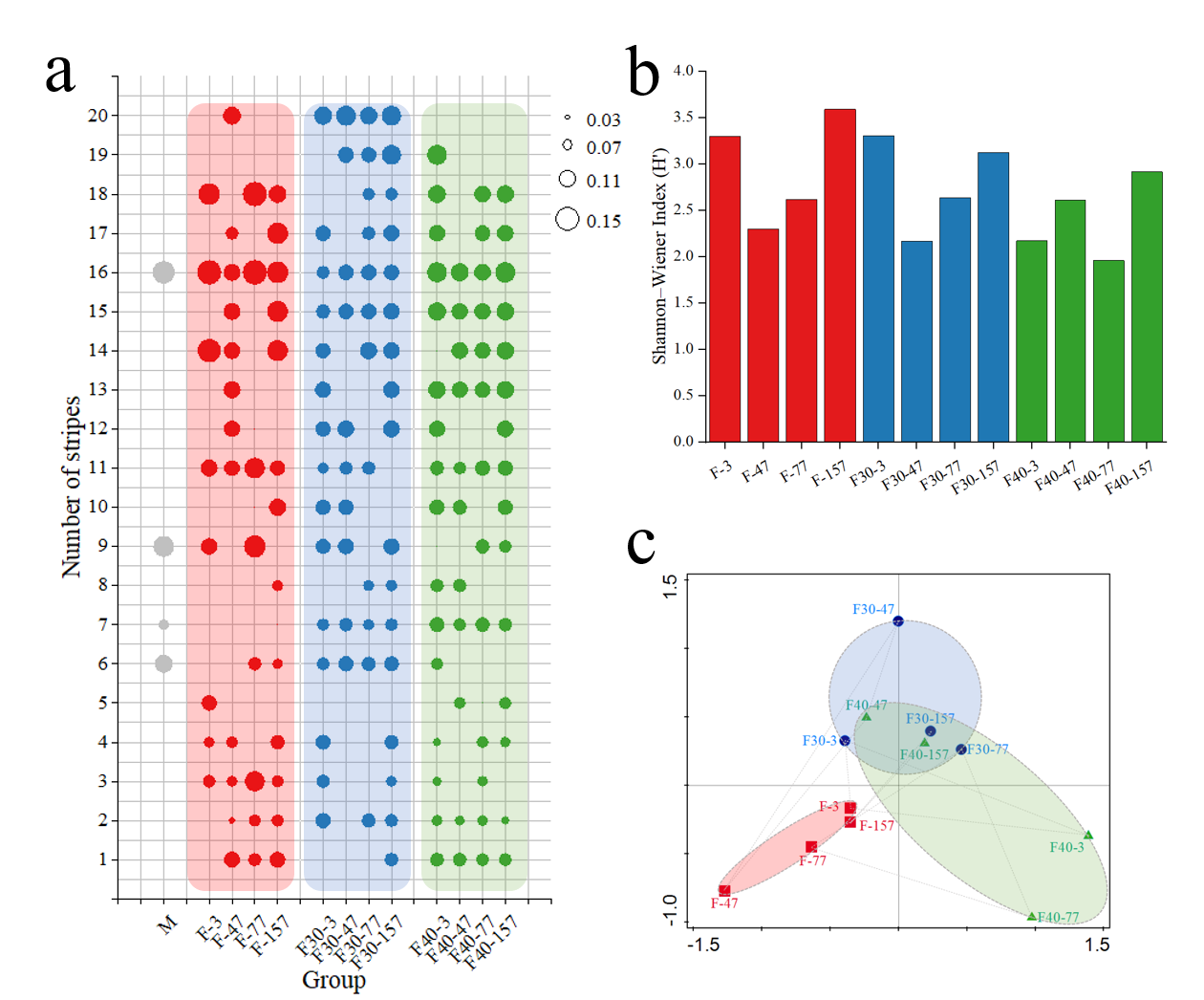


Figure S1. DGGE fingerprint (a), Shannon Wiener index (b), and NMDS analysis (c) in different treatments.
